# Clinical Advances in Cystic Fibrosis

**DOI:** 10.3390/jcm11216306

**Published:** 2022-10-26

**Authors:** Esther Imperlini, Rosanna Papa

**Affiliations:** 1 Department for Innovation in Biological, Agro-Food and Forest Systems, University of Tuscia, 01100 Viterbo, Italy; 2 Department of Public Health and Infectious Diseases, Sapienza University, p.le Aldo Moro 5, 00185 Rome, Italy

Over recent decades, significant advances have been achieved in ameliorating clinical outcomes for patients with cystic fibrosis (CF). The improvements in CF diagnosis and therapeutic strategies facilitated the transition from a life-shortening disease to a treatable chronic one [1]. Many CF-related studies were published in the last few years, highlighting that the scientific community is focused on increasing both quality of life and life expectancy for patients with CF [2].

CF, first described in 1938, is a recessive genetic disorder currently affecting over 100,000 individuals worldwide [3]. It is caused by mutations in the cystic fibrosis transmembrane conductance regulator (CFTR) gene, encoding for a chloride and bicarbonate ion channel, expressed at the surface of several epithelial cells [4]. Given the regulatory importance of this ion transport, dysfunction in the CFTR protein or its absence results in dehydration of the epithelial surface, with the consequent production of thick and sticky mucus, the retention of which leads to the obstruction, infection and inflammation of affected organs (especially the lungs, pancreas, gastrointestinal tract, liver and reproductive system) [5]. CF is a multiorgan disorder with wide-ranging clinical manifestations and ethnicity-related prevalence, although the most compromised organ is the lung, in which recurrent infections and local airway inflammation are the main cause of CF morbidity and mortality [6]. It is most common in Caucasian people and in Europe (with an incidence of 1:3000 births), whereas in African and Asian populations, the birth prevalence is lower, at 1:15,000 and 1:30,000, respectively [7]. The relatively high incidence of CF among Europeans may be related to the increased number (>2000) of CFTR genetic variants reported to date [8]; however, only about 500 variants have been recognized as responsible for causing this disease [9].

Despite most CFTR mutations being potentially pathogenic and many remaining still to be functionally characterized, about 80% of patients with CF carry one 508Fdel mutation and 40–50%, instead, are homozygous for this deletion [1].

Moreover, grouping the CFTR mutation into six functional classes (I–VI) according to their clinical consequences and their effect on CFTR function was undoubtedly useful: classes I–III contain CFTR mutations that are disease-causing and associated with severe phenotype but do not synthesize CFTR (I), have defective CFTR folding and trafficking (II), or have defective channel gating (III), respectively; in contrast, mutations in classes IV–VI are associated with a milder phenotype due to the maintenance of CFTR residual function despite a decreasing in-anion conductance (IV), in-protein abundance (V) or stability (VI) [1].

It is globally recognized that there is no one-to-one relationship between mutations and classification, since a single mutation can impair CFTR function via multiple molecular factors [10]. However, an advantage of this classification is that different mutations within the same class could be treated with the same therapeutic approaches.

There is no treatment for CF, and its management was traditionally based on therapies able to control and/or ease respiratory, digestive, inflammatory and infectious symptoms, thus preventing or reducing complications [10].

Since the identification of the CFTR gene in 1989, over recent decades, we have seen remarkable advances in the understanding of CF that, coupled with technological innovations, have contributed to prolonging life expectancy for patients with CF: among these, the number of adults is growing, and the current average age at survival is <50 years [1].

This significant increase in expectancy and quality of life can surely be attributed to (i) the improved early diagnosis, particularly newborn screening and genetic testing; (ii) the availability of therapies, including mucolytics, anti-inflammatory, antibiotics against infections, nutritional support and/or lung transplantation; and (iii) the multidisciplinary care team approach [1].

Although about 60% of patients with CF are adults in countries with well-established diagnosis and care, their survival and quality of life remain still limited with significant clinical, psychosocial and economic burdens for them and their caregivers.

Since 2012, new promising therapeutic tools have been introduced, and these include small molecules targeting either the CFTR or the cascade of events downstream from CFTR dysfunction. So-called CFTR modulators have been developed in order to potentiate, correct, stabilize or amplify CFTR functionality [11]. To date, there are four CFTR modulator drugs approved by the Food and Drug Administration (FDA) and the European Medicines Agency (EMA), but they are used in clinical practice only for specific genotypes and, as a consequence, for a small CF sub-population [9].

Despite the improved outcomes in patients with CF treated with the approved modulators, particularly related to lung function, there are many CFTR mutations without available and appropriate modulators. Although a growing number of patients with CF are becoming eligible for the CFTR modulators used in clinical practice, a survival gap in CF still remains to be bridged; this will be possible through the rationale design of highly effective CFTR modulators.

Currently, in addition to the use of modulators, research aims to reduce the impact of chronic infections affecting the respiratory system.

The most common cause of chronic infection in people with CF is *Pseudomonas aeruginosa*, up to a maximum of 80% of infected patients in adulthood [12]. *P. aeruginosa* is a virulent bacterium, the main pathogenic characteristics of which are related to its surface structures, secreted compounds and biofilm formation [13].

The sophisticated host–pathogen interactions that occur in acute and chronic pulmonary CF infections are the result of the tremendous adaptive ability of *P. aeruginosa* [14]. The initial survival in acute infection is favored by an arsenal of virulence factors and antibiotic resistance mechanisms. Additional adaptation, accomplished by a shift toward a more persistent sessile phenotype of *P. aeruginosa*, makes non-motile and slow-growing colonies with a tendency to form mucoid biofilms responsible for the immune evasion; colonization; and consequently, chronic infection [15]. Thus, despite the hostile lung environment in CF, *P. aeruginosa* can prosper, causing acute and chronic infections [16] and increasing the complexity of clinical management [17]. Mucoid biofilm formation permits *P. aeruginosa* to also colonize medical equipment, facilitating its diffusion among patients with CF. Furthermore, the recurrent administration of antibiotics increases the risk of acquiring multi-drug resistant (MDR) *P. aeruginosa*. This pathogen possesses a strong ability to develop natural and acquired antimicrobial resistance through genotypic or phenotypic modifications [18]. For this reason, the development of an anti-*P. aeruginosa* therapy should aim to target the specific resistance mechanism.

Despite intense efforts, the development of new antibiotics or therapeutic strategies effective against *P. aeruginosa* infections is very difficult due to the variability and complexity of antimicrobial resistance mechanisms, as well as the lack of an in-depth understanding of its pathogenicity. Designing effective therapeutic approaches (including phage therapy, immunotherapy, gene editing therapy, antimicrobial peptides and vaccine therapies) to counteract *P. aeruginosa* infections is increasing in urgency and requires collective efforts [19].

An attractive alternative for patients with CF could be the development of vaccines against *P. aeruginosa* [20,21]. However, despite over 50 years of research, clinical vaccine development has been largely unsuccessful [22]. In fact, there are no ongoing clinical trials investigating vaccines against *P. aeruginosa*.

Luckily, new CFTR modulators are currently under investigation, either in experimental development or in earliest phase trials [23]. In this scenario, a pivotal role is played by the development of preclinical cell models used for investigating in vitro efficacy and for predicting the in vivo therapeutic responsiveness of new potential drugs, targeting dysfunction associated with CFTR mutations, and those still uncharacterized or rare. This will lead not only to a comprehensive understanding of the molecular basis underlying CF but also to precision medicine, through which each individual may benefit from personalized therapy.
